# Characterization and Statistical Optimization of Enterobatin Synthesized by *Escherichia coli* OQ866153

**DOI:** 10.1007/s10528-023-10626-z

**Published:** 2024-01-21

**Authors:** Mohamed T. Khazaal, Ahmed H. I. Faraag, Marwa A. Hamada, Hoda H. El-Hendawy

**Affiliations:** Botany and Microbiology Department, Faculty of Science, Helwan University, Helwan, Cairo, 11795 Egypt

**Keywords:** Enterobactin biosynthesis genes, *Escherichia coli*, Siderophores, Statistical optimization

## Abstract

Microorganisms produce siderophores, which are secondary metabolites with a high affinity for iron. Siderophores have received significant attention due to their diverse applications in ecological and clinical research. In this study, siderophores production by *Escherichia coli* OQ866153 was optimized using two-stage statistical approach involving Plackett–Burman design (PBD) and response surface methodology (RSM) using central composite design (CCD). Out of 23 variables, succinate, tryptophan, Na_2_HPO_4_, CaCl_2_, agitation, and KH_2_PO_4_ were found to have the most significant effect on siderophores production in the first optimization stage with the highest SU% of 43.67%. In the second stage, RSM using CCD was utilized, and the optimal conditions were determined to be 0.3 g/l succinate, 0 g/l tryptophan, 6 g/l Na_2_HPO_4_, 0.1 g/l CaCl_2_, 150 RPM agitation, and 0.6 g/l KH_2_PO_4_, resulting in a maximum siderophore units (SU%) of 89.13%. The model was significant, as indicated by the model *f*-value of 314.14 (*p*-value = 0.0004) and coefficient of determination *R*^2^ of 0.9950. During validation experiments, the obtained maximum SU% was increased up to 87.1472%, which was two times as the value obtained under ordinary conditions (46.62%). The produced siderophores were purified and characterized using ^1^H, ^13^C NMR, IR spectroscopy. The obtained results indicated that the compound was enterobactin and *entABCDEF* genes were further detected in *Escherichia coli* OQ866153 extracted DNA. To our knowledge, this is the first report of statistical optimization for enterobactin synthesis by an *E. coli* strain isolated from a clinical source in Egypt.

**Supplementary Information:**

The online version contains supplementary material available at 10.1007/s10528-023-10626-z.

## Introduction

Iron (Fe), a key component of several fundamental cellular processes, is the fourth most ample element in Earth’s crust (Soares 2022). However, due to its low bioavailability, it may be a rare element. In order to alleviate Fe depletion, microorganisms and plants biosynthesis secrete and reuptake certain organic secondary metabolites with low molecular masses (500–1500 Da) called siderophores that specifically chelate Fe (III) ions (Soares 2022). More than 500 siderophores have been reported, of which several hundreds have been purified and structurally characterized (using HPLC, NMR, IR, and Mass-Spectroscopy), and continually new structures are discovered (Gama et al. 2021). Siderophores are typically categorized by the functional groups with which they bind iron. Catecholate, hydroxamate, and α-hydroxycarboxylate are the three main bidentate ligands. Mixed siderophores (pyoverdines) have multiple ligand types. On siderophores, ligands may have a linear or cyclic arrangement. Because cyclization improves complex stability, cyclic siderophores are more stable than linear complexes (Hofmann et al. 2020).

Siderophores and their substituted derivatives have a large number of applications in medical and environmental sciences. In agriculture, various types of siderophores have been found to act as a biological control agent against harmful phytopathogens and enhance the growth of several plant species (Molnár et al. 2023). They can also be used for detoxifying heavy-metal-contaminated samples, making them useful in bioremediation (Gupta et al. 2023). Furthermore, siderophores have the ability to detect iron content in various environments, serving as a biosensor (Khasheii et al. 2021). In the medical field, siderophores use the "Trojan horse strategy" to form complexes with antibiotics, enabling selective delivery of antibiotics to antibiotic-resistant bacteria (Rayner et al. 2023). Siderophores can also be utilized in the treatment of certain iron overload diseases, such as sickle cell anemia (Ribeiro et al. 2022). Siderophores can also be used to treat malaria, get rid of transuranic elements from the body (Handore et al. 2022), and have anticancer activity (Pita-Grisanti et al. 2022).

To achieve the given wide range of applications, it is essential to optimize siderophores production by studying the factors that influence their production. Concentration of siderophores may extensively vary in the culture medium, depending on the bacteria, culture medium, cultural conditions, iron-phosphorous concentrations, carbon–nitrogen sources, and the presence of metals (Soares 2022). Various experimental designs can be employed to optimize cell media for growth and production. Statistical experimental designs (such as full or partial factorial designs, Latin rectangles, central composite, and Plackett–Burman) offer a methodical and effective approach to conduct experiments to achieve specific objectives while simultaneously studying multiple factors in a short period of time and comprehending the interactions between various variables (Sahu and Prakash 2021).

The production of most siderophores has a significant challenge due to the presence of enantiomorphs, limiting steps of the chromophore synthesis, non-proteinogenic amino acids, and their molecular complexity. Therefore, this study aimed to optimize and characterize the siderophore production by *E. coli* strain OQ866153 through two-stage statistical approach based on Plackett–Burman design (PBD) and response surface methodology (RSM) via central composite design (CCD). The genes responsible for biosynthesis of the produced siderophore was also investigated.

## Materials and Methods

### The used Bacterial Strain

*E. coli* 49 was kindly provided by Professor Mona Mabrouk, National Organization for Drug Control and Research, Giza, Egypt. In a previous study, this strain was found to be antibiotic resistant, and siderophores producer and its identity was confirmed as an *E. coli* strain by using 16S rRNA sequence analysis (Khazaal et al. 2022). Recently, the sequence was deposited at the National Center for Biotechnology Information (NCBI) database with an accession numbers OQ866153.

### Quantitative Determination of Siderophores

Siderophores production by *E. coli* OQ866153 was previously determined (Khazaal et al. 2022) using Chrome Azurol Sulfonate (CAS) broth assay of Alexander and Zuberer (1991) after growth in M9 medium at 37 °C for 24 h. Bacterial cells were then removed by centrifugation at 6000 rpm at room temperature for 10 min. The supernatant was filter sterilized through 0.45 µm membrane filter and then added to CAS assay solution which consisted of [Solution 1; 21.9 mg of hexadecyltrimethylammonium bromide (HDTMA) in 25 ml of double-distilled water, 1.5 ml of 1 mM FeCl_3_.6H_2_O in 10 mM HCl, 7.5 ml of 2 mM CAS, Solution 2; 9.76 g of 2-[N-morpholino] ethanesulfonic acid (MES buffer) was dissolved in 50 ml and the pH adjusted to 5.6 using 50% KOH)] in (1:1) and mixed with shuttle (87.3 mg of 5-sulfosalicylic acid) solution (100:1). The obtained blue mixture was incubated in the dark for 15 min at room temperature, after which the degree of color change was measured using T60 UV–Vis Spectrophotometer (PG Instruments LTD) at 630 nm. Siderophores concentration in the culture supernatant was calculated and expressed as siderophores unit percent (SU%) using the following formula: [SU% = (*A*_r_−*A*_s_/*A*_r_) × 100], where Ar is the reference solution absorbance and *A*_s_ is the sample solution absorbance, at 630 nm.

### Optimization of Siderophores Production

#### Plackett–Burman Design (PBD) for Selecting Significant Variables

Twenty-three independent variables were used in PBD; four of them were temperature, incubation time, pH, and agitation as physical variables, eighteen chemical variables were used as glycerol, glucose, glutamate, succinate, NH_4_Cl, (NH_4_)_2_SO_4_, urea, asparagine, phenylalanine, tyrosine, tryptophane, KH_2_PO_4_, K_2_HPO_4_, MgSO_4_, FeSO_4_, NaCl, Na_2_HPO_4_, and CaCl_2_, and one dummy variable. Two levels (low (− 1) and high (+ 1)) of each variable, as indicated in Table 1, were submitted to the Design Expert version 13 program, and twenty-five trials were obtained (Table 2). The PB runs were carried out in duplicate, and the produced siderophores were determined quantitatively as siderophores unit percent (SU%) by CAS shuttle assay according to Alexander and Zuberer (1991). PBD depends on the first-order model *Y* = β0 + Σ*βiXi* where *Y* represents the siderophores unit percent (SU%), while *β*0 denotes the model intercept, βi represents the linear coefficient, and *X*_*i*_ indicates the level of the independent variable.

**Table 1:** Experimental variables and their levels for Plackett–Burman (PB) design

Number	Variable	Code	Unit	−1	+ 1
1	Temperature	A	°C	28	37
2	Incubation Time	B	H	24	48
3	pH	C	N	5	8
4	Agitation	D	RPM	50	150
5	Glycerol	E	ml/l	1	10
6	Glucose	F	g/l	1	5
7	Glutamate	G	g/l	0	1
8	Succinate	H	g/l	0.3	2
9	NH_4_Cl	J	g/l	0.1	1
10	(NH_4_)_2_SO_4_	K	g/l	0.1	1
11	Urea	L	g/l	0	1
12	Asparagine	M	g/l	1	5
13	Phenylalanine	N	mg/l	0	1
14	Tyrosine	O	mg/l	0	1
15	Tryptophane	P	mg/l	0	1
16	KH_2_PO_4_	Q	g/l	0.6	6
17	K_2_HPO_4_	R	g/l	0.4	4
18	MgSO_4_	S	g/l	0	1
19	FeSO_4_	T	mM	0.2	2
20	NaCl	U	g/l	0	0.5
21	Na_2_HPO_4_	V	g/l	0	6
22	CaCl_2_	W	g/l	0	0.1
23	Dummy	X	N	− 1	1

The numbers from 1 to 23 represent the different physical and chemical variables with their corresponding codes, units, low level (− 1), and high level (+ 1) for PB-design

*°C* celsius degree, *H* hours, *N* nothing, *RPM* revolutions per minute, *ml/l* milliliter per liter, *g/l* gram per liter, *mg/l* milligram per liter, *mM* milli-Mollar

**Table 2:** The trials and SU% response obtained from Plackett–Burman design

Trials	A	B	C	D	E	F	G	H	J	K	L	M	N
1	28	24	8	50	10	1	0	0.3	0.1	1	1	5	1
2	37	48	5	50	10	1	1	0.3	0.1	0.1	0	5	1
3	37	48	5	150	1	5	1	0.3	0.1	1	1	1	0
4	37	48	5	50	10	5	0	0.3	1	0.1	1	1	0
5	28	24	5	150	10	5	1	2	0.1	1	0	5	1
6	37	24	8	50	1	1	0	2	1	1	1	5	0
7	28	48	5	150	10	1	0	2	1	0.1	0	5	0
8	37	24	8	150	1	1	1	2	0.1	0.1	1	1	1
9	32.5	36	6.5	100	5.5	3	0.5	1.15	0.55	0.55	0.5	3	0.5
10	28	48	5	150	1	1	0	0.3	1	1	1	5	1
11	37	48	8	150	1	5	0	2	1	0.1	0	5	1
12	28	48	8	150	10	5	0	2	0.1	1	1	1	0
13	28	24	8	150	1	1	1	0.3	1	0.1	0	1	0
14	28	48	8	50	1	5	1	0.3	0.1	1	0	5	0
15	37	48	8	150	10	1	1	0.3	1	1	0	1	1
16	28	24	5	50	10	5	1	2	1	0.1	1	1	1
17	28	24	8	150	10	5	1	0.3	1	0.1	1	5	0
18	28	48	8	50	1	5	0	2	0.1	0.1	0	1	1
19	37	48	8	50	10	1	1	2	0.1	0.1	1	5	0
20	28	24	5	50	1	1	0	0.3	0.1	0.1	0	1	0
21	37	24	5	150	1	5	0	0.3	0.1	0.1	1	5	1
22	37	24	8	50	10	5	0	0.3	1	1	0	1	1
23	37	24	5	150	10	1	0	2	0.1	1	0	1	0
24	28	48	5	50	1	1	1	2	1	1	1	1	1
25	37	24	5	50	1	5	1	2	1	1	0	5	0

Trials	O	P	Q	R	S	T	U	V	W	X	SU%
1	1	0	6	0.4	1	2	0	0	0.1	1	12.75
2	1	1	6	0.4	1	0.2	0.5	6	0	− 1	21.55
3	1	0	6	0.4	0	0.2	0	6	0.1	1	26.59
4	0	0	6	4	1	2	0.5	0	0.1	− 1	13.51
5	0	0	6	4	0	0.2	0.5	0	0.1	− 1	4
6	1	0	6	4	0	0.2	0.5	6	0	− 1	12.84
7	1	0	0.6	0.4	0	2	0.5	6	0.1	1	6.21
8	0	0	0.6	0.4	1	2	0.5	6	0.1	− 1	34.35
9	0.5	0.5	3.3	2.2	0.5	1.1	0.25	3	0.05	0	27.7
10	0	1	0.6	4	1	0.2	0	6	0.1	− 1	17.14
11	0	0	6	0.4	1	0.2	0	0	0	1	12.61
12	1	1	0.6	0.4	1	0.2	0.5	0	0	− 1	6.44
13	1	1	6	4	1	0.2	0.5	0	0.1	1	26.78
14	0	0	0.6	4	1	2	0.5	6	0	1	43.67
15	1	0	0.6	4	0	2	0	0	0	− 1	41.53
16	1	0	0.6	4	1	0.2	0	6	0	1	14.8
17	0	1	6	0.4	0	2	0	6	0	− 1	31.09
18	1	1	6	4	0	2	0	6	0.1	− 1	8.98
19	0	1	0.6	4	0	0.2	0	0	0.1	1	5.32
20	0	0	0.6	0.4	0	0.2	0	0	0	− 1	38.51
21	1	1	0.6	4	0	2	0.5	0	0	1	27.56
22	0	1	0.6	0.4	0	0.2	0.5	6	0.1	1	13.73
23	0	1	6	4	1	2	0	6	0	1	19.08
24	0	1	6	0.4	0	2	0.5	0	0	1	0
25	1	1	0.6	0.4	1	2	0	0	0.1	− 1	9.5

*A* temperature, *B* incubation time, *C* pH, *D* agitation, *E* glycerol, *F* glucose, *G* glutamate, *H* succinate, *J* NH_4_Cl, *K* (NH4)_2_SO_4_, *L* urea, *M* asparagine, N phenylalanine, *O* tyrosine, *P* tryptophane, *Q* KH_2_PO_4_, *R* K_2_HPO_4_, *S* MgSO_4_, *T* FeSO_4_, *U* NaCl, *V* Na_2_HPO_4_, *W* CaCl_2_, *X* dummy variable, *SU%* siderophores unit percent

#### Optimization of Significant Variables Using Response Surface Methodology (RSM)

One of the RSM is the central composite design in which the interactions between the significant factors obtained from PBD results were studied (Korany et al. 2021). In this investigation, six variables (succinate, tryptophane, Na_2_HPO_4_, CaCl_2_, agitation, and KH_2_PO_4_) at five levels (-alpha (-α), low (−1), middle (0), high (+1) and + alpha (+*α*)) were used (Table 3). Fifty-five trials were obtained, the experiments done in duplicate with fixing the remained variables at their low levels and siderophores unit percent (SU%) was obtained as a response (Table 4). The following polynomial second-order equation was used to fit the experimental results: *Y* = *β*_0_ + ∑ *β*_*i*_ *X*_*i*_ + ∑ *β*_*ii*_ *X*_*i*_^2^ + ∑ *β*_*ij*_ *X*_*i*_ *X*_*j*_ where *Y* is the predicted response, *β*_0_ is the regression coefficients, *β*_*i*_ is the linear coefficient, *β*_*ii*_ is the quadratic coefficients, *β*_*ij*_ is the interaction coefficients, and *X*_*i*_ is the coded levels of independent variable.

**Table 3:** Independent variables and their levels for central composite Design

NO	Variables	Code	Units	Range and levels				
				−α*	−1	0	+ 1	+ α*
1	Succinate	A	g/l	0.3	0.725	1.15	1.575	2
2	Tryptophane	B	mg/l	0	0.25	0.5	0.75	1
3	Na_2_HPO_4_	C	g/l	0	1.5	3	4.5	6
4	CaCl_2_	D	g/l	0	0.025	0.05	0.075	0.1
5	Agitation	E	RPM	50	75	100	125	150
6	KH_2_PO_4_	F	g/l	0.6	1.95	3.3	4.65	6

^*^1.68179

**Table 4:** Trials and results for central composite Design

Run	Variables						SU% response
	A	B	C	D	E	F	
1	1.15	0.5	3	0.05	100	3.3	54.1673
2	1.15	0.5	3	0.05	100	3.3	55.1412
3	1.15	0.5	3	0.05	100	3.3	53.1483
4	1.15	0.5	3	0.05	100	3.3	55.1542
5	1.15	0.5	3	0.05	100	3.3	52.1589
6	1.15	0.5	3	0.05	100	3.3	54.1621
7	1.575	0.5	3	0.05	100	3.3	49.129
8	1.575	0.5	3	0.05	100	3.3	48.103
9	1.15	0.5	3	0.05	100	3.3	55.1532
10	1.15	0.5	3	0.05	100	3.3	53.663
11	1.15	0.5	3	0.05	100	3.3	55.8468
12	0.3	0	6	0	50	0.6	88.6281
13	0.3	0	6	0	150	6	87.8545
14	0.3	1	0	0.1	150	0.6	62.6319
15	0.3	0	6	0.1	50	6	65.1572
16	0.3	1	0	0.1	50	6	41.5765
17	0.3	1	6	0.1	50	0.6	62.7671
18	0.3	0	0	0	50	6	64.1769
19	0.3	0	0	0.1	50	0.6	62.8185
20	0.3	1	6	0.1	150	6	61.9584
21	0.3	0	0	0	150	0.6	87.2843
22	0.3	0	6	0.1	150	0.6	89.1311
23	0.3	1	0	0	50	0.6	59.7972
24	0.3	0	0	0.1	150	6	61.1099
25	0.3	1	6	0	150	0.6	84.224
26	0.3	1	0	0	150	6	62.1871
27	0.3	1	6	0	50	6	64.1254
28	0.725	0.5	3	0.05	100	3.3	60.6859
29	0.725	0.5	3	0.05	100	3.3	61.6598
30	2	0	6	0.1	50	6	36.1169
31	2	1	0	0	150	6	35.4743
32	2	0	6	0	150	6	54.332
33	2	1	6	0.1	150	6	37.2084
34	2	1	6	0	150	0.6	54.0384
35	2	1	6	0	50	6	37.3348
36	2	0	0	0	150	0.6	53.0862
37	2	1	0	0.1	50	6	21.5459
38	2	1	0	0.1	150	0.6	32.2063
39	2	0	0	0.1	150	6	35.2474
40	2	0	0	0	50	6	33.3738
41	2	0	0	0.1	50	0.6	34.1026
42	2	0	6	0	50	0.6	55.1522
43	2	1	6	0.1	50	0.6	34.0637
44	2	0	6	0.1	150	0.6	52.9958
45	2	1	0	0	50	0.6	34.331
46	1.15	0.5	3	0.05	100	4.65	50.6639
47	1.15	0.5	3	0.025	100	3.3	54.8133
48	1.15	0.5	3	0.05	75	3.3	51.6208
49	1.15	0.5	4.5	0.05	100	3.3	53.0435
50	1.15	0.5	3	0.05	100	1.95	54.7028
51	1.15	0.75	3	0.05	100	3.3	50.3526
52	1.15	0.5	1.5	0.05	100	3.3	51.3642
53	1.15	0.25	3	0.05	100	3.3	52.0557
54	1.15	0.5	3	0.075	100	3.3	50.5832
55	1.15	0.5	3	0.05	125	3.3	56.774

*A* succinate, *B* tryptophane, *C* Na_2_HPO_4_, *D* CaCl_2_, *E* agitation, *F* KH_2_PO_4_, *SU%* siderophores unit percent

#### Validation of Central Composite Design

**Fig. 1:**
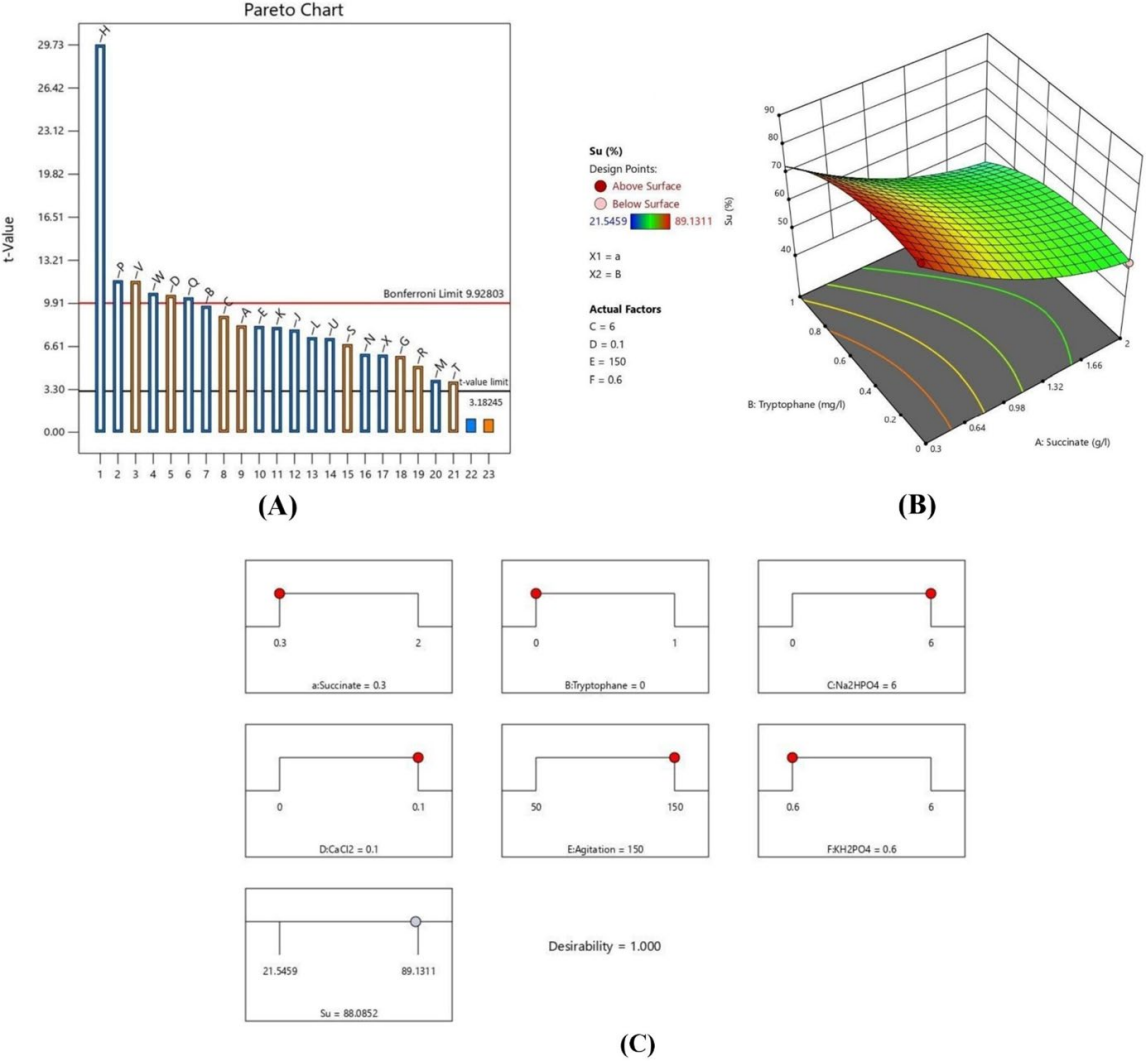
Optimization of siderophores production by *E. coli* OQ866153. **a** Pareto chart showing the most significant independent variables on siderophores production. *A* Temperature, *B* incubation time, *C* pH, *D* agitation, *E* glycerol, *F* glucose, *G* glutamate, *H* succinate, *J* NH_4_Cl, *K* (NH_4_)_2_SO_4_, *L* urea, *M* asparagine, *N* phenylalanine, *O* tyrosine, *P* tryptophane, *Q* KH_2_PO_4_, *R* K_2_HPO_4_, *S* MgSO_4_, *T* FeSO_4_, *U* NaCl, *V* Na_2_HPO_4_, *W* CaCl_2_, *X* dummy factor. **b** Three-dimensional graph (3D) for the most significant interaction between succinate (**a**) and tryptophan (**b**) showed the highest predicted SU% ~ 88.13%. **c** The model solution that actually validated with the highest SU% = 87.15%

Once the data were analyzed, the model was tested for its adequacy and precision. The software solution shown in Fig. 1c was applied in duplicate.

### Statistical Analysis

Design-expert 13 software (Stat-Ease Inc. Minneapolis, USA) was used for the experimental design and data analysis.

#### Siderophores Biomass Production

According to Fiedler et al. (2001), siderophores from *E. coli* OQ866153 strain were extracted as follows: in an Erlenmeyer flask, 100 ml of M9 growth medium was inoculated with OQ866153 strain and incubated overnight at 37 °C and 150 rpm. Then, 10 ml of previously prepared culture was added to one liter of the same medium and incubated for 24 h at 37 °C and 150 rpm with shaking. After incubation, the bacterial suspension was centrifuged at 4 °C and 10,000 rpm for 15 min using a Sigma 3-16PK centrifuge (Germany) and the supernatant was collected and filter sterilized using 0.45 µl Millipore cellulose nitrate membrane filter (CHM, Spain). The filtrate pH was adjusted to 3 using concentrated 12 N hydrochloric acid, then one liter ethyl acetate was added for 1 h at 10 °C and 100 rpm in a shaking incubator. Finally, the ethyl acetate extract was evaporated using a rotary evaporator (IKA, Germany) at 40 °C to obtain a dry crude and stored at − 20 °C until further use.

#### Extraction and Purification of Siderophore

By using a silica gel thin-layer chromatography (TLC) plate (Merck-Germany) and a Dichloromethane (DCM): Methanol (9:1 v/v) as a solvent system, the siderophore compound was extracted as follows: in a chromatographic jar was initially equilibrated with the solvent system for 20 min, the TLC plate that loaded with ethyl acetate extract was subsequently immersed in the solvent system to facilitate complete elution and separation of compounds. Then, the TLC plate was carefully removed and visualized under a UV lamp (VL-215 LC, Marne La Vallee, France) at 254/365 nm. The desired band that showed a bright blue fluorescence, was carefully scratched, transferred to another vial, and eluted several times using ethyl acetate to obtain the siderophore compound. This process was repeated multiple times to obtain the desired quantity of the compound, which was then purified using a sephadex LH-20 column and a DCM: Methanol (6:4 v/v) solvent system.

### Characterization of the Purified Siderophore Compound

#### Nuclear Magnetic Resonance (NMR) Spectroscopy

The purified compound was characterized by ^1^H and ^13^C NMR spectroscopy (Moreno et al. 2018) using a Bruker Corp. spectrophotometer with frequencies of 400 and 100 MHz at the NMR Lab in the Microanalytical Unit (MAU) of the Faculty of Pharmacy at Cairo University, Egypt.

#### Fourier Transform Infrared (FTIR) Spectroscopy

The IR spectrum of the purified compound (Moreno et al. 2018) was performed using PerkinElmer L1600400 FTIR spectrum (UK) within a wavelength range and a resolution of 400–4000 cm^−1^ and 4 cm^−1^, respectively, at the central lab, Faculty of Science, Helwan University, Cairo, Egypt.

#### Detection of Enterobactin Biosynthesis Genes

The primers of enterobactin biosynthesis genes (*entA, entB, entC, entD, entE*, and *entF*) were designed using OLIGO 7.57 software (Molecular Biology Insights, Inc) and the *E. coli* strain NZ_CP050203 sequence was used as a reference **(**Table 7**).** Bacterial DNA was extracted following the DNA QIAamp mini kit (QIAGEN) instructions. After DNA extraction, the designed primers were used for the PCR amplification of the enterobactin biosynthesis genes and the PCR conditions were denaturation at 95 °C/5 min, 30 denaturation cycles at 94 °C/45 s, annealing at 59 °C/30 s, extension at 72 °C/30 s, and a final 72 °C/5 min extension step. The amplification products were separated by 1% agarose gel electrophoresis and visualized under UV transilluminator. Finally, the PCR products of enterobactin biosynthesis genes were sequenced using their forward primers in an automatic 3730XL ABI prism sequencer (Applied Biosystems, USA) at Korea Macrogen Inc. and Geneious prime 2021 software was employed to evaluate the BLAST results and build phylogenetic tree with the corresponding genes in gene bank database.

## Results

### Optimization of Siderophores Production

#### PB- Design Results

Results of PB-design (25 runs) revealed that the percentage of siderophores unit (SU%) increased from 0 (run 24), 8.98% (Run 18), 13.73 (Run 22), 26.78% (Run 13), and 34.35% (Run 8), to the highest value 43.67% in run 14 as indicated in Table 2. Furthermore, the model *p*-value was 0.0094 (< 0.05), indicating its significance. The incubation temperature (A), incubation time (B), pH (C), agitation (D), glycerol (E), glutamate (G), succinate (H), NH_4_Cl (J), (NH_4_)_2_SO_4_ (K), urea (L), phenylalanine (N), tryptophane (P), KH_2_PO_4_ (Q), K_2_HPO_4_ (R), MgSO_4_ (S), NaCl (U), Na_2_HPO_4_ (V), and CaCl_2_ (W) variables were found to be with significant *p*-values of 0.0146, 0.0105, 0.0123, 0.0089, 0.0147, 0.0281, 0.0011, 0.0157, 0.015, 0.0183, 0.057, 0.0266, 0.0073, 0.0092, 0.0367, 0.0186, 0.0074, and 0.0087, respectively. The model *Adeq* precision ratio of 43.0822 indicated an adequate signal, where a ratio greater than 4 is desirable, and confirmed that the model can be used to navigate the design space (Table 5). Additionally, as in the Pareto chart (Fig. 1a), the variables succinate (H), tryptophan (P), Na_2_HPO_4_ (V), CaCl_2_ (W), agitation (D), and KH_2_PO_4_ (Q) were deemed the most significant on the production of siderophores, with *f*-values of 883.61, 135.63, 134.5, 114.06, 110.73, and 106.98, respectively.

**Table 5:** Statistical results (ANOVA) for Plackett–Burman and central composite designs

ANOVA of PBD				
Variables	Coefficients	Mean square	*f*-value	*p*-value
A-temperature	0.2699	1.75	66.99	0.0146
B-time	− 0.3197	2.45	94.00	0.0105
C-pH	0.2940	2.07	79.51	0.0123
D-Agitation	0.3470	2.89	110.73	0.0089
E-Glycerol	− 0.2685	1.73	66.30	0.0147
G-Glutamate	0.1926	0.8905	34.13	0.0281
H-Succinate	− 0.9802	23.06	883.61	0.0011
J-NH_4_Cl	− 0.2596	1.62	62.00	0.0157
K-(NH_4_)_2_SO_4_	− 0.2659	1.70	65.03	0.0150
L-Urea	− 0.2403	1.39	53.10	0.0183
M-Asparagine	− 0.1321	0.4190	16.06	0.0570
N-Phenylalanine	− 0.1983	0.9435	36.16	0.0266
P-Tryptophane	− 0.3840	3.54	135.63	0.0073
Q-KH_2_PO_4_	− 0.3410	2.79	106.98	0.0092
R-K_2_HPO_4_	0.1673	0.6715	25.73	0.0367
S-MgSO_4_	0.2226	1.19	45.58	0.0212
T-FeSO_4_	0.1281	0.3937	15.09	0.0603
U-NaCl	− 0.2381	1.36	52.15	0.0186
V-Na_2_HPO_4_	0.3824	3.51	134.50	0.0074
W-CaCl_2_	− 0.3522	2.98	114.06	0.0087
X-Dummy	− 0.1964	0.9259	35.48	0.0270

Model statistics				
Standard error	*R*^2^	*Adeq* precision	Model *f*-value	Model *p*-value
0.0330	0.9991	43.0822	106.32	0.0094

ANOVA of CCD				
Variables	Coefficients estimate	Standard error	*f*-value	*p*-value*
A-Succinate	− 14.34	0.5580	660.11	0.0023
A^2^	5.44	3.79	2.06	0.2020
B-Tryptophane	− 5.41	0.2462	483.69	< 0.0001
C-Na_2_HPO_4_	5.69	0.2462	534.61	< 0.0001
D-CaCl_2_	− 5.13	0.2462	435.10	< 0.0001
E-Agitation	4.88	0.2462	392.41	< 0.0001
F-KH_2_PO_4_	− 4.63	0.2462	353.88	< 0.0001
AB	1.21	0.2481	23.75	< 0.0001
AC	− 0.6371	0.2481	6.60	0.0169
AD	0.5466	0.2481	4.85	0.0375
AE	− 0.5865	0.2481	5.59	0.0266
AF	0.9310	0.2481	14.09	0.0010
BC	− 0.3812	0.2481	2.36	0.1376
BD	0.3017	0.2481	1.48	0.2359
BE	− 0.2227	0.2481	0.8062	0.3783
BF	0.7244	0.2481	8.53	0.0075
CD	− 0.2443	0.2481	0.9701	0.3346
CE	0.0279	0.2481	0.0126	0.9115
CF	− 0.1671	0.2481	0.4536	0.5071
DE	− 0.2256	0.2481	0.8273	0.3722
DF	0.2152	0.2481	0.7525	0.3944
EF	− 0.3742	0.2481	2.27	0.1447
B^2^	− 6.50	3.62	3.21	0.0851
C^2^	− 2.50	3.62	0.4750	0.4970
D^2^	−0.5200	3.62	0.0206	0.8870
E^2^	5.48	3.62	2.28	0.1433
F^2^	− 0.5796	3.62	0.0256	0.8742

Model statistics				
Standard error	Coefficient of variation (CV) %	*R*^2^	Model *f*-value	Model *p*-value
0.4974	2.91	0.9950	314.14	0.0004

*p ≤ 0.05–significant at 5% level

#### CC- Design Results

Results of CC-design shown in Table 4 demonstrated that the interaction between the most significant variables of PB-design increased the SU% from 21.54% (Run 37), 37.33% (Run 35), 49.12 (Run 7), 56.77% (Run 55), and 64.12% (Run 27) to the highest value 89.13% (Run 22).CCD data analysis demonstrated the model *p*-value of 0.0004 and *f*-value of 314.14, indicating its significance. The coefficients for the linear terms were succinate (-14.34), tryptophan (-5.41), Na_2_HPO_4_ (5.69), CaCl_2_ (-5.13), Agitation (4.88), and KH_2_PO_4_ (-4.63); the interactive coefficients for the two-factor interactions were AB (1.21), AC (-0.6371), AD (0.5466), AE (-0.5865), AF (0.9310), BC (-0.3812), BD (0.3017), BE (-0.2227), BF (0.7244), CD (-0.2443), CE (0.0279), CF (-0.1671), DE (-0.2256), DF (0.2152), and EF (-0.3742), and the quadratic coefficients for A^2^, B^2^, C^2^, D^2^, E^2^, and F^2^ were 5.44, -6.50, -2.50, -0.52, 5.48, and -0.5769, respectively (Table 5). The model determination (R^2^) coefficient of 0.9950 indicated that it could explain approximately 99.50% of the variability in the response. The results of the model also determined the impact of each input parameter on siderophore production by applying the following actual second-order polynomial equation.

$$\begin{array}{lllll}
R\left( {{\rm{SU }}\% } \right) =  & 53.7237 - 14.3362*{\rm{succinate}} - 5.4138*{\rm{tryptophane}} + 5.69163*{\rm{N}}{{\rm{a}}_{\rm{2}}}{\rm{HP}}{{\rm{O}}_4} - 5.1347*{\rm{CaC}}{{\rm{l}}_2}\\
 & \; + 4.87627*{\rm{agitation}} - 4.63072*{\rm{K}}{{\rm{H}}_2}{\rm{P}}{{\rm{O}}_4} + 1.20902*{\rm{succinate}}*{\rm{tryptophane}} - 0.63715*{\rm{succinate}}*{\rm{N}}{{\rm{a}}_{\rm{2}}}{\rm{HP}}{{\rm{O}}_4}\\
 & \; + 0.5466*{\rm{succinate}}*\;{\rm{CaC}}{{\rm{l}}_2} - 0.58645*{\rm{succinate}}*{\rm{agitation}} + 0.93105*{\rm{succinate}}*{\rm{K}}{{\rm{H}}_{\rm{2}}}{\rm{P}}{{\rm{O}}_{\rm{4}}}\\
 & \; - 0.381194*{\rm{tryptophane}}*{\rm{N}}{{\rm{a}}_{\rm{2}}}{\rm{HP}}{{\rm{O}}_4} + 0.301706*{\rm{tryptophane}}*{\rm{CaC}}{{\rm{l}}_{\rm{2}}} - 0.222744*{\rm{tryptophane}}*{\rm{agitation}}\\
 & \; + 0.724419*{\rm{tryptophane}}*{\rm{K}}{{\rm{H}}_{\rm{2}}}{\rm{P}}{{\rm{O}}_4} - 0.244344*{\rm{N}}{{\rm{a}}_{\rm{2}}}{\rm{HP}}{{\rm{O}}_{\rm{4}}}*{\rm{CaC}}{{\rm{l}}_2} + 0.0278813{\rm{ * N}}{{\rm{a}}_{\rm{2}}}{\rm{HP}}{{\rm{O}}_{\rm{4}}}{\rm{* glycerol}}\\
 & \; - 0.167081*{\rm{N}}{{\rm{a}}_{\rm{2}}}{\rm{HP}}{{\rm{O}}_4}*{\rm{K}}{{\rm{H}}_{\rm{2}}}{\rm{P}}{{\rm{O}}_{\rm{4}}} - 0.225644*{\rm{CaC}}{{\rm{l}}_2}*{\rm{agitation}} + 0.215194*{\rm{CaC}}{{\rm{l}}_2}*{\rm{K}}{{\rm{H}}_2}{\rm{P}}{{\rm{O}}_4}\\
 & \; - 0.374156*{\rm{agitation }}*{\rm{K}}{{\rm{H}}_{\rm{2}}}{\rm{P}}{{\rm{O}}_{\rm{4}}} + 5.43712*{\rm{succinate}}*{\rm{succinate}}\; - 6.49644*{\rm{tryptophane}}*{\rm{tryptophane}}\\
 & \;\; - 2.49764*{\rm{N}}{{\rm{a}}_{\rm{2}}}{\rm{HP}}{{\rm{O}}_4}*{\rm{N}}{{\rm{a}}_{\rm{2}}}{\rm{HP}}{{\rm{O}}_{\rm{4}}}{\rm{\;}} - 0.520039*{\rm{CaC}}{{\rm{l}}_2}*{\rm{CaC}}{{\rm{l}}_2}\; + 5.47656*{\rm{agitation}}*{\rm{agitation}}\; - 0.579639*{\rm{K}}{{\rm{H}}_2}{\rm{P}}{{\rm{O}}_4}*
\end{array}$$

#### Interactions Between the Significant Variables

The interactions between the six significant variables for maximum siderophore production were studied through three dimensional plots. Different significant interactions were observed between the variables AB, AC, AD, AE, AF, and BF. The most significant one was between succinate and tryptophan (AB), where, at a constant level of Na_2_HPO_4_ (*C* = 6 g/l), CaCl_2_ (*D* = 0.1 g/l), agitation (*E* = 150 rpm), and KH_2_PO_4_ (*F* = 0.6 g/l), the presence of the lowest or highest tryptophan (B) levels with low level of succinate (A) resulted in a higher siderophore production (SU%) of 88.1 and 72.8%, respectively. On the other hand, siderophore production (SU%) lessened to 53.8 and 43.3% in the presence of the lowest or highest tryptophan (B) levels with high level of succinate (A), respectively (Fig. 1b).

The model was validated using one of the suggested solutions and the results showed SU% of 87.1472%, which deviated by − 1% from the predicted value. Nonetheless, this value was approximately doubled compared to the SU% obtained before optimization, which was 46.62%.

#### Extraction, Purification, and Characterization of the Synthesized Siderophores

**Fig. 2:**
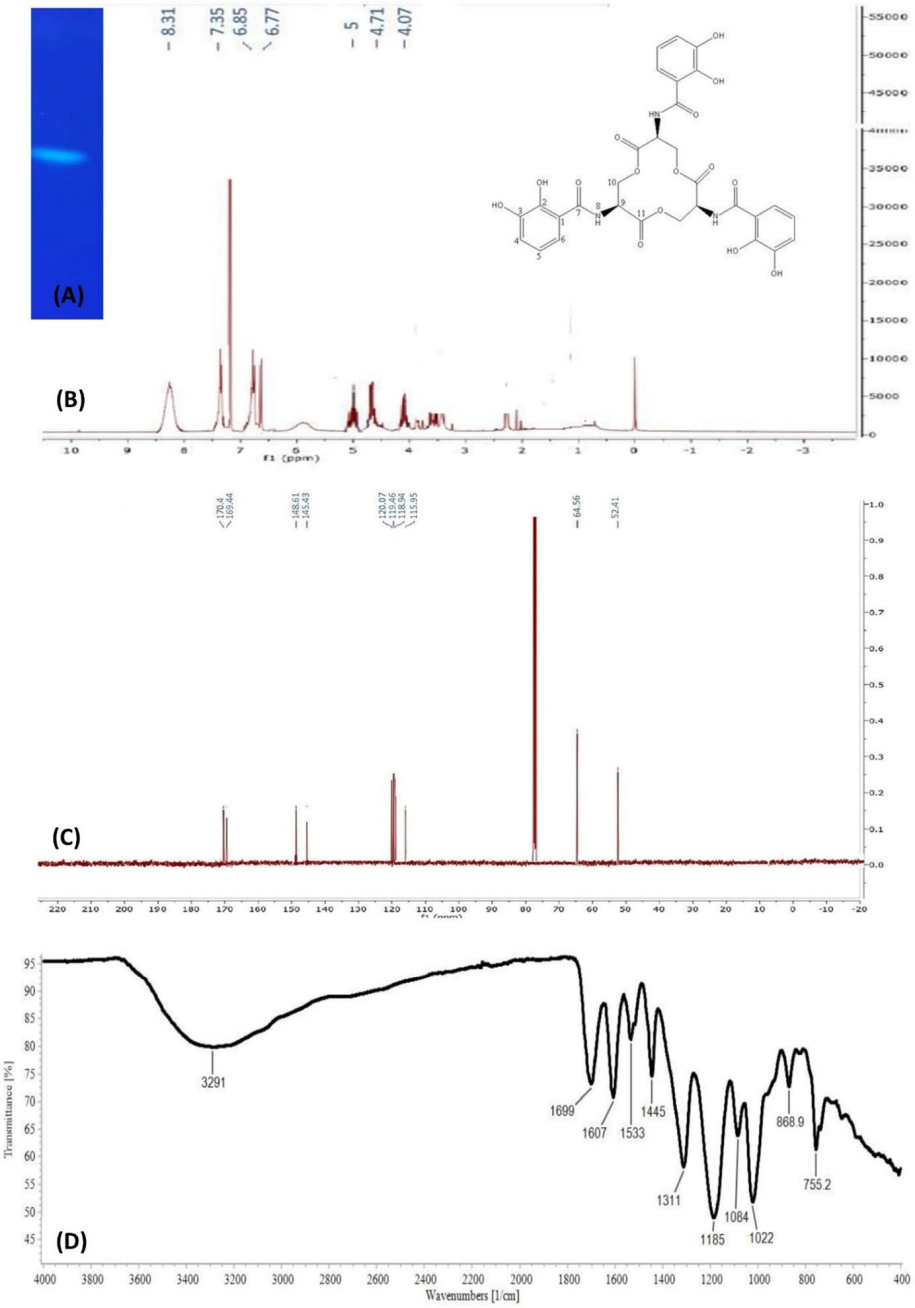
Characterization of enterobactin from *E. coli* OQ866153**. a** A bright blue fluorescence band of enterobactin after purification. **b–d** ^1^H, ^13^C NMR and FTIR of enterobactin, respectively

*E. coli* OQ866153 strain was grown in M9 medium under the optimum growth condition. The synthesized siderophores were extracted and separated on TLC plates then examined under the UV lamp at 254/365 nm which showed a bright blue fluorescent band (Fig. 2a). This band was scratched and eluted, and the compound was characterized by using NMR (^1^H and ^13^C) and FTIR spectroscopy. The ^1^H NMR spectrum indicated the presence of proton chemical shift signals at δ 4.07, 4.71, 5.00, 6.77, 6.85, 7.35, and 8.31 ppm (Fig. 2b). Similarly, the ^13^C NMR spectrum recorded 10 different carbon signals at δ 52.41, 64.56, 115.95, 118.94, 119.46, 120.07, 145.43, 148.61, 169.44, and 170.40 ppm (Fig. 2c). The chemical shifts were compared with those reported in (Table 6). On the other hand, the results of FTIR revealed a broad band located at the wavenumber of 3000–3500 cm^−1^, which corresponded to the overlap between OH and NH (Fig. 2d). Additionally, the band observed at 1690 cm^−1^ could correspond to the amide-carbonyl group's characteristic. The bands at 1607 and 1532 cm^−1^ represented the C=C stretch and C3–H bend overlaps. The following IR region (1000 –1300 cm^−1^) was associated with C–OH, C–H, N–H, and OH. The results obtained indicated that the compound was enterobactin.

**Table 6:** ^13^C and ^1^H chemical shift values (ppm) of enterobactin compared to other reported NMR spectrum data

Atoms	Obtained enterobactin		Moreno et al. (2018)	
	^13^C	^1^H	^13^C	^1^H
	Solvent: CDCl_3_		Solvent: CD_3_OD	
1(C	115	–	116.8	–
2(C–OH)	148.6	–	149.7	–
3(C–OH)	145.4	–	147.2	–
4(CH)	120.1	6.85(dd, J = 8.5,2 Hz)	119.8	6.94
5(CH)	119.5	6.77(dd, J = 8.5, 8.5 Hz)	119.6	6.69
6(CH)	118.9	7.35(dd, J = 8.5,2 Hz)	119.7	7.24
7(C = O)	170.4	–	170.7	
8(NH)	–	8.31	–	–
9(CH)	52.4	5.00(m)	53.7	5.04
10(CH_2_)	64.6	4.07(dd, J = 11.5,5.9) 4.71(dd, J = 11.5,3.5)	65.8	4.61 4.69
11(C = O)	169.4	–	–	

(–) Not detected

#### Detection of Enterobactin Biosynthesis Genes

**Fig. 3:**
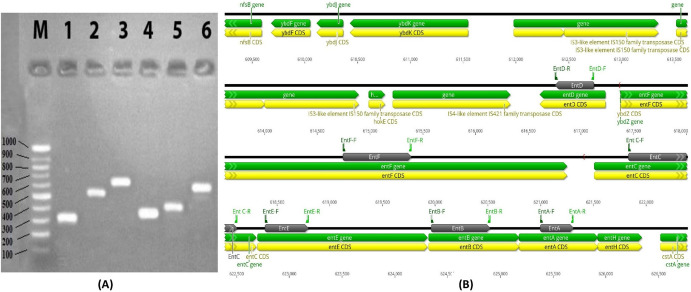
**a** Agarose gel electrophoresis of amplified *entABCDEF* genes of *E. coli* OQ866153. Lanes M, 100 bp molecular weight DNA marker; *1 entA* OR645470*, 2 entB* OR645471, *3 entC* OR645472, *4 entD* OR645473, *5 entE* OR645474, *6 entF* OR645475. **b** BLAST Hit of sequenced enterobactin production genes (black color) with *E. coli* NZ_CP050203

**Fig. 4:**
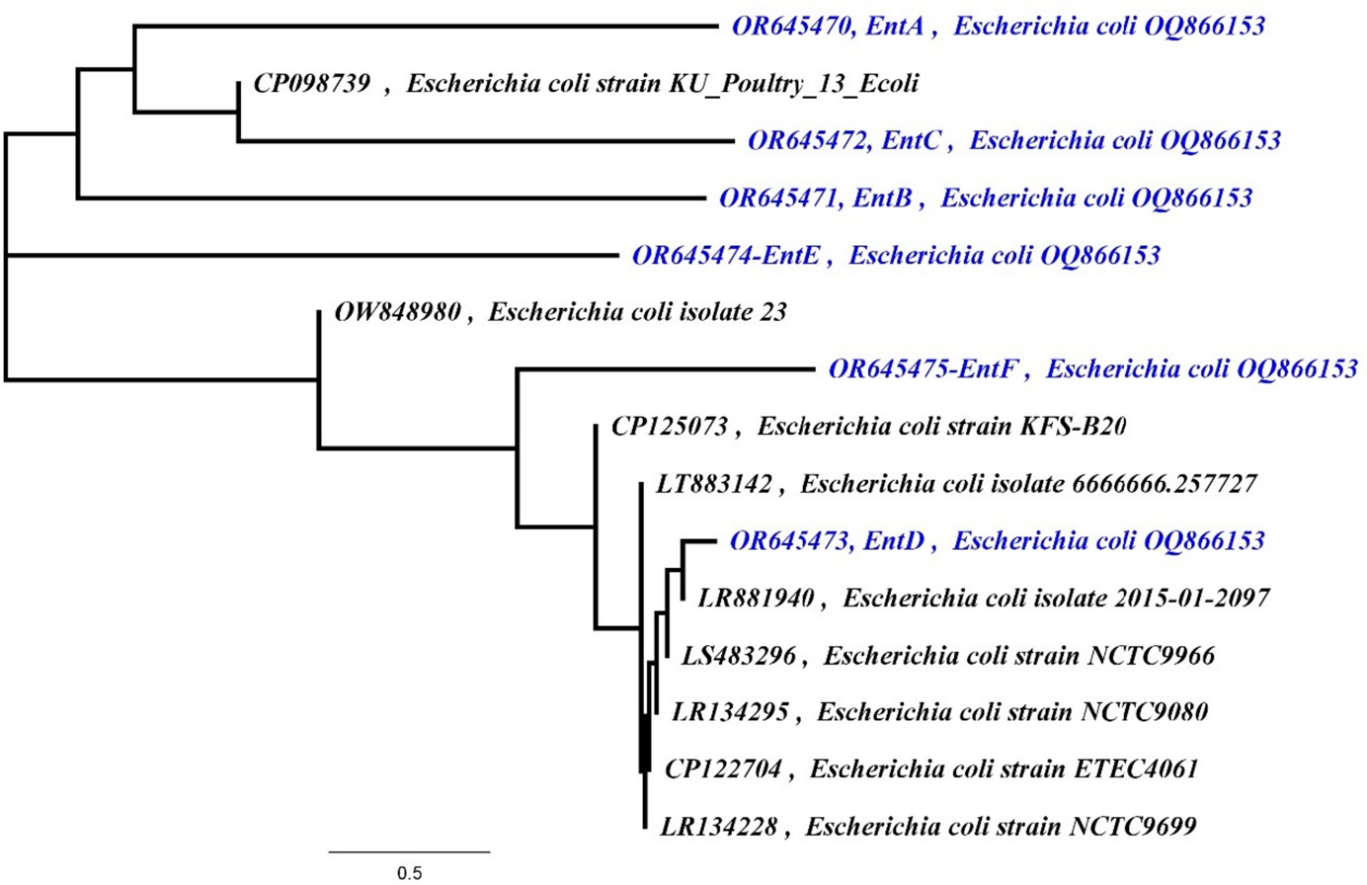
Phylogenetic analysis of enterobactin synthesis genes (*entA* OR645470, *entB* OR645471, *entC* OR645472, *entD* OR645473, *entE* OR645474, and *entF* OR645475)

**Fig. 5:**
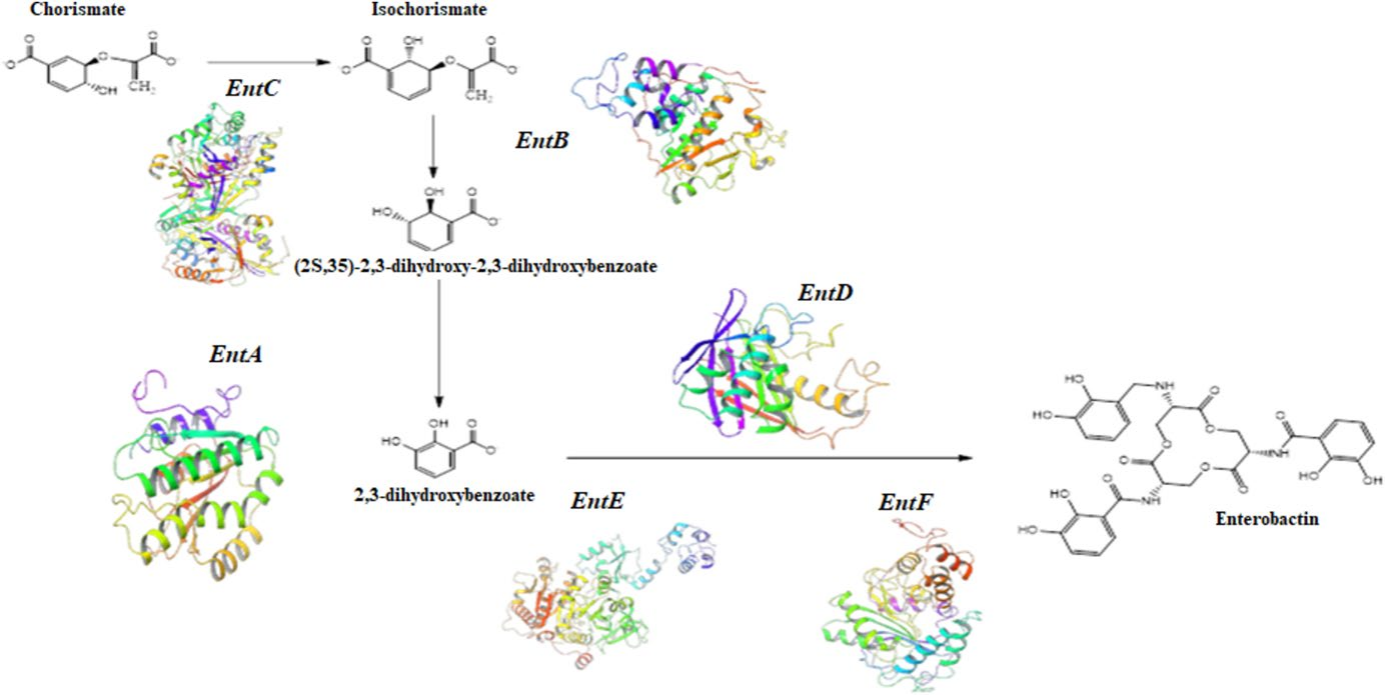
Biosynthetic pathway of enterobactin

Enterobactin designed primers were amplified using PCR for detection of enterobactin biosynthesis genes in *E. coli* OQ866153 extracted DNA and the amplicons were examined using agarose gel electrophoresis. Six bands corresponding to enterobactin biosynthesis (*entA, entB, entC, entD, entE,* and *entF*) genes with different sizes (Table 7) were detected in the gel (Fig. 3a). These bands were excised, eluted, purified, and sequenced by the sequencing services of Macrogen in Korea (Fig. S1). The obtained sequences were submitted separately to the gene bank and the accession numbers provided were *entA*: OR645470, *entB*: OR645471, *entC*: OR645472, *entD*: OR645473, *entE*: OR645474, and *entF*: OR645475. The sequenced DNA were then subjected to a Blast hit analysis against *E. coli* RH-045-MS chromosome (NZ_CP050203), as shown in Fig. 3b, where the sequenced genes were highlighted in black. The sequence alignment showed > 90% identity percentage to the corresponding genes in the gene bank (Table S1) and represented by phylogenetic tree (Fig. 4). These results indicated that the peptidic assembly of enterobactin could be attributed to the non-ribosomal peptide synthetases (*entABCDEF*) as demonstrated in Fig. 5.

**Table 7:** Designed primers and PCR products size of enterobactin synthesis genes

Gene	PCR product size	Primer	Primer	Ta
*entA*	314	203F19	5′ TGAAACGGAGCGACTGGAC 3′	60.0 °C
		516R20	5′ AAACCACATTACAGCGCACG 3′	
*entB*	560	25F21	5′ TACGCACTGCCGGAGTCTCAC 3′	60.0 °C
		563R22	5′ CCGGCCACATATTTCAGCGACA 3′	
*entC*	671	322F21	5′ CGTTTTACCCGCAGCCAGTCG 3′	60.7 °C
		972R21	5′ ACAATGCCGCCAAACAGTTCG 3′	
*entD*	369	110F21	5′ CACAACTGCAACACGCTGGAC 3′	58.6 °C
		455R24	5′ TCTCACTTGCCTTAAATGCGCTCT 3′	
*entE*	411	76F21	5′ CCACTGACCGACATTCTGACT 3′	59.0 °C
		466R21	5′ GTTATGCTCACCGCTGTCGTT 3′	
*entF*	648	1762F21	5′ CCGCTGCAACTTTCACAACCG 3′	60.4 °C
		2389R21	5′ ATAGAGATCACCCGCCACACC 3′

## Discussion

Siderophores could be applied as biosensors, biocontrol, bioremediation, and chelation agents, in addition to weathering soil minerals and improving plant growth, (Ahmed and Holmström 2014; Khasheii et al. 2021).

For future applications of siderophores produced by *E. coli* OQ866153, statistical optimization of siderophores production by this strain was carried out. Different cultural conditions and physiological variables were used to optimize siderophores production. Two-step approaches were used in the optimization experiment: the first one was Plackett–Burman (PB) to recognize the most significant factors, and the second was central composite design (CCD) to determine the optimal concentration of selected variables and assess their interactions. The siderophores unit percent (SU%) was used as the response (Y) to maximize siderophore production in each trial. Gao and Bian (2022) suggested that the production of siderophores in microorganisms is influenced by the type of substrate used. As a result, a total of 23 independent variables were selected based on a component of M9 medium, succinate medium (Shaikh et al. 2016), and enterobactin biosynthesis pathway on Kegg pathway under accession number (Entry: map01053) in Plackett–Burman design experiment. The PB and CC models were both statistically significant, with *p*-values of 0.0094 and 0.0004, respectively. Pham et al. (2019) noted that smaller *p* -values and larger *f* -values represent the significance of the corresponding coefficients. PB results showed that Run 14 recorded the highest siderophores percentage (SU%) of 43.67% and revealed that the following variables succinate, tryptophan, Na_2_HPO_4_, CaCl_2_, KH_2_PO_4_, and agitation were the most significant. These results nearly agree with Kalyan et al. (2022) as they mentioned that the organic and inorganic nutrients present in the medium are significant predictors of siderophore production in a given organism. Different nitrogen sources, including NH_4_Cl, (NH_4_)_2_SO_4_, urea, phenylalanine, tryptophane, and asparagine, were tested and the results revealed that tryptophane was found to be the most effective with a *p* -value of 0.0073. These results agreed with Mushtaq et al. (2022) who found that L-tryptophane improves iron uptake in *Acinetobacter calcoaceticus* and *Bacillus simplex*, resulting in better plant growth and not agreed with Sayyed et al. (2019) who reported that in the presence of urea, both *Pseudomonas fluorescence* and *Pseudomonas putida* exhibited maximum productivity of siderophores. Also, Srivastava et al. (2022) found that (NH_4_)_2_ SO_4_, NaNO_3_, and urea facilitate siderophore production with a SU value larger than 60%. The choice of carbon source is critical within the components of the culture medium (Vindeirinho et al. 2021). In this study, succinate as a carbon source was the most significant one (with *f*-value of 883.61) comparing with the other tested sources. Our findings were in consistent with those described that *Pseudomonas fluorescens* DSM 50090 significantly produced higher pyoverdine in minimal medium containing succinate than that with dextrose or glycerol (Vindeirinho et al. 2021). Furthermore, phosphorus is crucial in culture media because it is necessary for cellular metabolism and can act as a buffer. In the *Pseudomonas* genus, it has been reported that the concentration of phosphate can impact PVD production (Soares 2022). One of the most important aspects of our study was agitation, which had a significant, positive impact on siderophore production. Our findings corroborated those of Panda et al. (2017), who found that siderophore production was too low when conducted under static conditions but optimal at 150 rpm. According to RajeNimbalkar et al. (2022), the determination coefficient (R^2^) value which was closer to 1, indicating the strength of the model and its ability to predict the response. In this study, the R^2^ values were 0.9991 and 0.995 in both PB and CC, indicating that 99.91 and 99.5% of the variability in siderophore production could be explained by the model’s independent variables, respectively. From PB results, six significant factors were introduced to central composite design to determine their optimal range and study the interaction between them. Ezemagu et al. (2021) reported that a high coefficient of variation (CV) indicated low model experiment reliability. The precision of the experiments in this study was demonstrated by a CV of 2.91%. CC results showed increase in SU% from 21.5 in run 37 to 89.1 in run 22. The results nearly matched with RajeNimbalkar et al. (2022), who reported that the maximum siderophore production was 98% in succinate medium during CC results. A 3D graphs was plotted to perform the variables interactions and the results showed that the most significant one was between succinate and tryptophan and the highest siderophore production (SU%) of 88.1 was obtained with the presence of the lowest level of tryptophan and succinate at a constant level of Na_2_HPO_4_ (*C* = 6 g/l), CaCl_2_ (*D* = 0.1 g/l), agitation (*E* = 150 rpm), and KH_2_PO_4_ (*F* = 0.6 g/l), indicating that presence of the two factors together had a negative effect on the siderophores production. These findings were in close agreement with those of Vindeirinho et al. (2021), who found that succinate performed best among the carbon sources tested; however, increasing succinate concentration in the culture medium did not lead to an increase in PVD maximum production, despite leading to a rise in biomass. The statistical model was validated under the predicted conditions (0.3 g/l succinate, 0 mg/l tryptophan, 6 g/l Na_2_HPO_4_, 0.1 g/l CaCl_2_, 150 rpm agitation, and 0.6 g/l KH_2_PO_4_ and the siderophore production (SU%) was 87.1472% increased by two-fold compared to the value obtained from the ordinary conditions (46.62% before optimization). The results were almost in agreement with those reported by Srivastava et al. (2022), who found that after siderophores were optimized by *Pseudomonas monteilii* strain MN759447, the actual values were 89.9% siderophore units, which was 10% higher than the value obtained during CAS quantification (80.06%). Moreover, the maximum production of a siderophore compound produced by an endophytic fungus *Talaromyces trachyspermus* was 88.9% through the application of Plackett–Burman design and Response Surface Methodology via Central Composite Design.

To confirm the structure of the extracted and purified compound, ^1^H, ^13^C NMR, and IR spectroscopy were utilized. The use of spectroscopy, specifically ^1^H and ^13^C NMR, is a common method for characterizing siderophore compounds due to the distinct features they provide (Fraga-Corral et al. 2022). NMR spectroscopy aids in identifying structural properties, specific functional groups present in the sample, and determining conformation and saccharide ratio within the mixture. The obtained carbon and proton chemical shifts were compared with previously reported data (Moreno et al. 2018). The ^1^H NMR spectrum displayed three doublet of doublet signals representing the three protons of the aromatic ring, with peaks at δ 6.77, 6.85, and 7.35 ppm for protons 5, 4, and 6, respectively. The coupling constant indicated that proton 5 was in the ortho position to both 6 and 4, while protons 4 and 6 were in the meta position to each other. An amide proton signal appeared at δ 8.31 ppm and a multiplate signal was observed at δ 5.00 ppm. Additionally, two doublet of doublet signals were detected for methylene protons at carbon 10, indicating the presence of enterobactin compound.

Based on the ^13^C NMR data, it was observed that there are 10 carbon signals present in the compound. Two of these signals, namely C7 and C11, were identified as characteristic carbonyl signals with a chemical shift of δ 170 and 169, respectively. Additionally, two oxygenated carbons, C3 and C2, were identified at δ 145.4 and 148.6, respectively. Furthermore, three aromatic methines were identified at δ 118.9, 119.5, and 120.1 for C6, C5, and C4, respectively. A quaternary carbon signal was observed at δ 115.9 for C1, while a methylene carbon signal was identified at δ 64.6 for C10. Lastly, a signal at δ 52.4 was observed for C9. By comparing this data with reported literature Moreno et al. (2018) and analyzing the ^1^H and ^13^C NMR data, it was concluded that the compound was enterobactin.

FTIR is used to differentiate between organic and inorganic materials and to acquire information about the functional groups present in certain compounds (Leela and Anchana 2017). For instance, the wide band observed at 3000–3500 cm^−1^ is frequently linked to the overlap between OH and NH, as noted by Subi et al. (2022). Moreno et al. (2018) found that this broad band was made up of three overlapping OH stretching modes, which were NH stretching bands at higher frequencies and showed three separate medium-sharp bands that each belonged to a different catechol amide arm. According to Kaur et al. (2021), proton transfer to neighboring oxygen may cause some shifts in N–H IR bands. The 1740 cm^−1^ band in the IR spectrum was assigned to represent the crown ester and amide group on carboxylic acid derivatives (Wang et al. 2022). Workman and Weyer (2012) reported that a more electronegative substituent can increase the carbonyl carbon oxygen stretch frequency above the nominal frequency of 1715 cm^−1^ by up to 100 cm^−1^. Carbonyl stretching bands were also observed at 1770 and 1818 cm^−1^ in studies of carboxylic acid derivatives and alpha-Angelica lactones (Moreno et al. 2018). However, in this study, the band was observed at 1690 cm^−1^. Moreno et al. (2018) also reported that the frequency of ester IR bands varies depending on whether it is in a crown ester, as in enterobactin, or in an open chain. According to Moreno et al. (2018), the bands observed at 1607 and 1532 cm^−1^ are attributed to the C=C stretch and C3–H bend overlaps. The C–OH, C–H, N–H, and OH stretching from catechol are observed in the 1000–1300 cm^−1^ IR region, which is consistent with Moreno et al. (2018) findings. By comparing the obtained IR spectroscopy data with the literature reported by Moreno et al. (2018), the compound was identified as enterobactin.

Enterobactin is a cyclic trimer of 2,3-dihydroxybenzoyl-l-serine produced by bacteria such as *Escherichia coli* and *Klebsiella pneumophila* belonging to family Enterobacteriaceae to scavenge Fe (III) with high affinity (Soares 2022). In this study, enterobactin encoding genes were detected in the DNA extracted from *E. coli* OQ866153 using enterobactin designed primer. The obtained results revealed that six bands corresponding to enterobactin biosynthesis (*entA* OR645470, *entB* OR645471, *entC* OR645472, *entD* OR645473, *entE* OR645474, and *entF* OR645475) genes with the size ranging from 314 to 671 bp were detected. The sequence alignment of the purified amplicons showed identity percentage higher than 90% to the corresponding genes in the gene bank. Most siderophores are produced by non-ribosomal peptide synthetases (NRPSs) and polyketide synthases (PKSs) working along with NRPS modules, but a small number are produced by other mechanisms (Kramer et al. 2020). In this study, the peptidic assembly of enterobactin could be attributed to the non-ribosomal peptide synthetases (*entABCDEF*), where enterobactin synthetase, a two-module NRPS, is responsible for *E. coli* siderophores biosynthesis and under iron-deficient conditions, the NRPS (*entB*, *entE*, and *entF)* components are induced for enterobactin synthesis (Liu et al. 2023). Reitz et al. (2017) reported that the protein products of the *entA, entB, entC, entD, entE,* and *entF* genes are 2,3-dihydro-2,3-dihydroxybenzoate dehydrogenase, isochorismatase, isochorimate synthase, 4-phosphatetheinyl transferase, 2,3-dihydroxybenzoyl adenylate synthase, and enterobactin non-ribosomal peptide synthetase, respectively. Enterobactin is synthesized through a two-step process, starting with the conversion of chorismic acid into isochorismate, followed by its conversion into 2,3-dihydro-2,3-dihydroxybenzoate, and finally to 2,3-dihydroxybenzoic acid (DHB). These successive steps are catalyzed by *entC, entB*, and *entA*, respectively (Reitz et al. 2017). *entD, entE, entF*, and a C-terminal aryl carrier of *entB* are involved in the formation of the amide linkage between DHB and l-serine (Jaremko et al. 2020). Before serine binds onto *entF* peptidyl carrier protein domain, it is activated by adenylation (Reitz et al. 2017). Finally, after the intermolecular cyclization and hydrolysis of three molecules of DHB-Ser, the *entF* terminal thioesterase domain releases enterobactin (Jelowicki and Butler 2022). According to the research by Jelowicki and Butler 2022), the strain of *Vibrio campbellii* BAA-1116 harbors a cluster of genes (*entA-F*) that are similar to the enterobactin biosynthetic gene cluster (BGC). Also, they suggested that AebABCE is responsible for the production of 2,3-dihydroxybenzoic acid (2,3-DHBA). Furthermore, (Wellawa et al. 2022) noticed that the *entABCDEF* operon proteins, synthesize and converge three *N*-2,3-dihyroxybenzoyl-l-serines to accommodate Fe^3+^. The antibacterial protein enterobactin shields *E. coli* from the oxidative stress caused by agents like hydrogen peroxide and several agricultural pesticides (Peralta et al. 2022). Due to its potent iron chelating characteristics, iron-free enterobactin is able to selectively induce lethal effects on highly proliferative cells. As a result, enterobactin shows promise as a powerful anticancer agent (Saha et al. 2019).

## Conclusion

In conclusion, using a two-stage statistical approach involving Plackett–Burman design (PBD) and response surface methodology (RSM) using central composite design (CCD) increased the production of siderophores by *E. coli* OQ866153. Out of 23 variables, succinate, tryptophan, Na_2_HPO_4_, CaCl_2_, agitation, and KH_2_PO_4_ were found to have the most significant effect on siderophores production in the first optimization stage with SU% response increased from 0 (Run 24) to the highest value of 43.67% (Run 14). In the second stage, CCD results showed increase in the SU% from 21.5 in run 37 to 89.1 in run 22., and the optimal conditions were determined to be 0.3 g/l succinate, 0 g/l tryptophan, 6 g/l Na_2_HPO_4_, 0.1 g/l CaCl_2_, 150 RPM agitation, and 0.6 g/l KH_2_PO_4_. Additionally, CCD results showed that the most significant variables interaction was between succinate and tryptophan with the highest siderophore production (SU%) of 88.1 in the presence of the lowest level of tryptophan and succinate at a constant level of Na_2_HPO_4_ (*C* = 6 g/l), CaCl_2_ (*D* = 0.1 g/l), agitation (*E* = 150 rpm), and KH_2_PO_4_ (*F* = 0.6 g/l), indicating that presence of the two factors together had a negative effect on the siderophores production. The statistical model was validated under the predicted conditions (0.3 g/l succinate, 0 mg/l tryptophan, 6 g/l Na_2_HPO_4_, 0.1 g/l CaCl_2_, 150 rpm agitation, and 0.6 g/l KH_2_PO_4_ and the siderophore production (SU%) was 87.1472% increased by two-fold compared to the value obtained from the ordinary conditions (46.62% before optimization). Based on ^1^H, ^13^C, and IR spectroscopy results, the purified siderophore was identified as enterobactin, a catecholate-type siderophore that is widely distributed among enteric bacteria. The presence of *entABCDEF* genes confirmed the biosynthetic pathway of enterobactin in *E. coli* OQ866153. To our knowledge, this is the first report of statistical optimization for enterobactin production by an *E. coli* strain isolated from clinical source in Egypt. This study provides valuable insights into the diversity and potential applications of siderophores in environmental and medical research.

## Supplementary Information

Below is the link to the electronic supplementary material.

Supplementary file1 (DOCX 601 kb)

## Data Availability

All data generated or analyzed during this study are included in this manuscript and its supplementary information files.
