# Supplementary material for: Characterization and Statistical Optimization of Enterobatin Synthesized by Escherichia coli OQ866153
Source: Biochem Genet. 2024 Jan 21;62(5):3920–45. doi: 10.1007/s10528-023-10626-z (PMC11427530; doi:10.1007/s10528-023-10626-z)
Supplement: Supplementary file 1 — Supplementary file1 (DOCX 601 kb) [file 10528_2023_10626_MOESM1_ESM.docx]

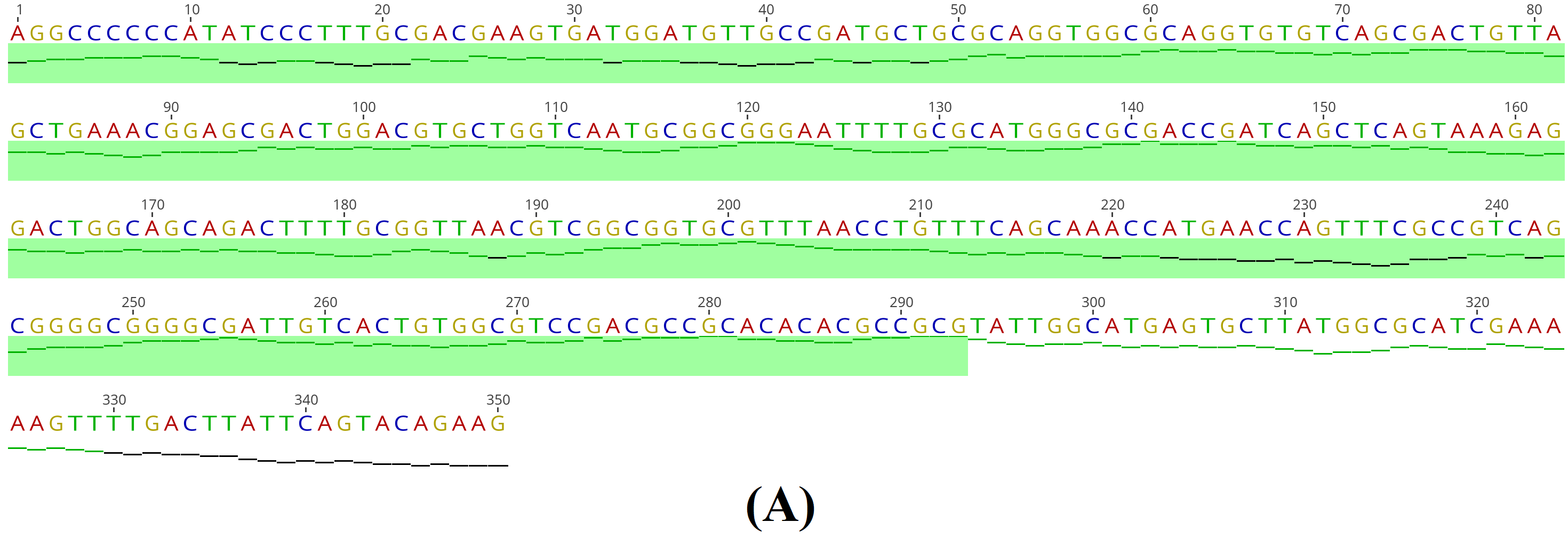


**Fig. S1.** Sequences of amplified enetrobactin synthesis genes of *E. coli* OQ866153**. (A)**; *EntA* OR645470*,* (**B);** *EntB* OR645471*,* **(C)**; *EntC* OR645472*,* **(D)**; *EntD* OR645473*,* **(E);** *EntE* OR645474*,* and **(F)** *EntF* OR645475 genes.


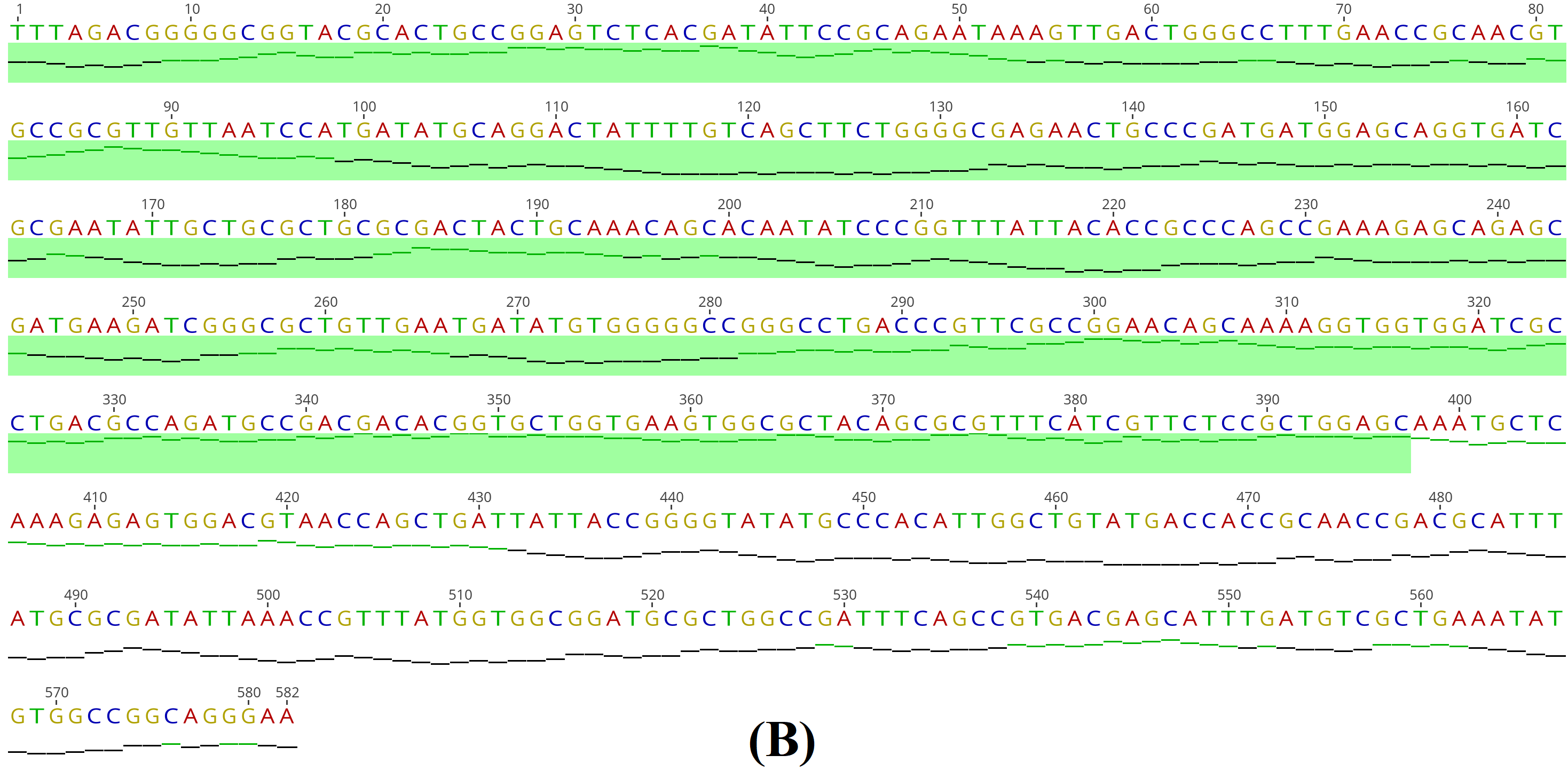


**Fig. S1.** Continued**.**


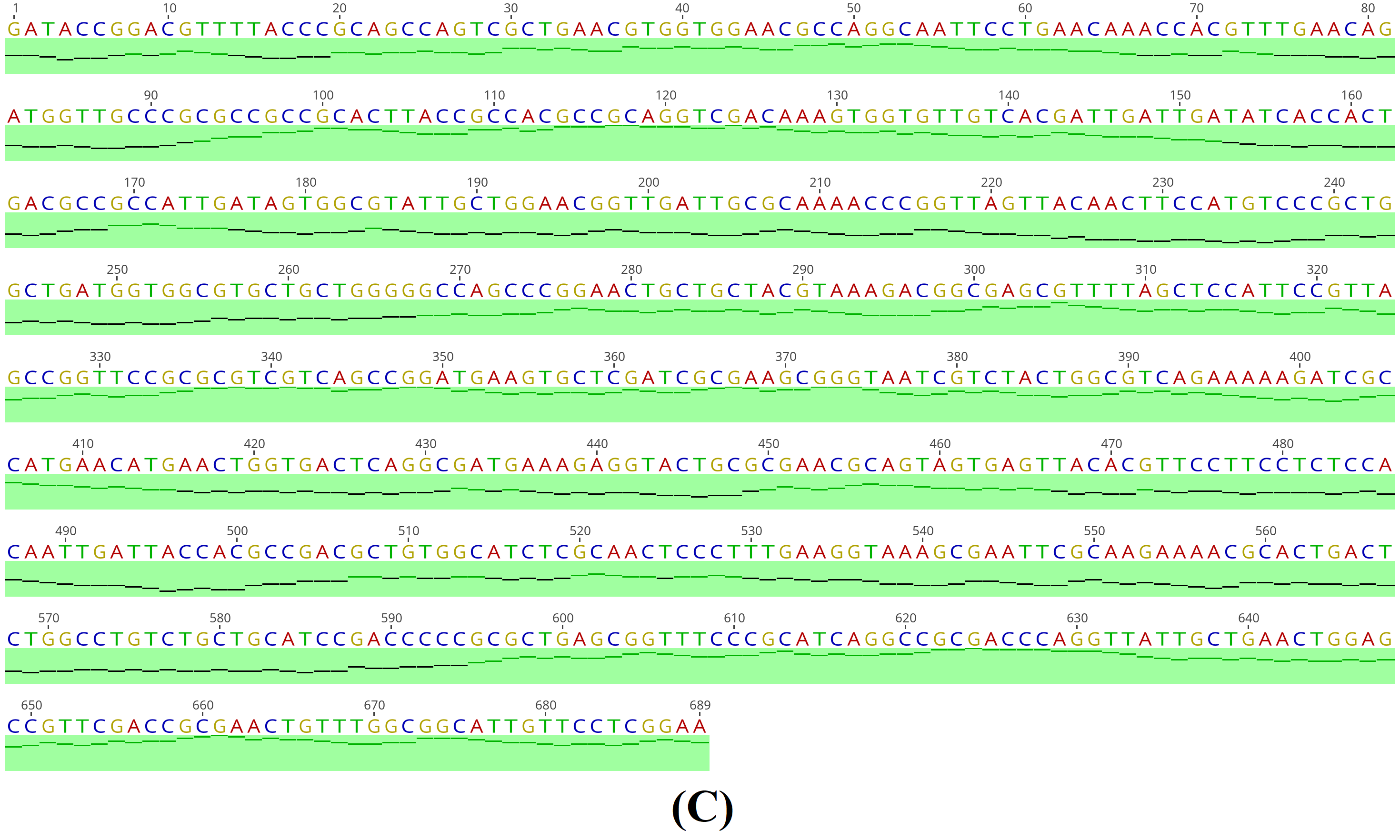
**Fig. S1.** Continued**.**


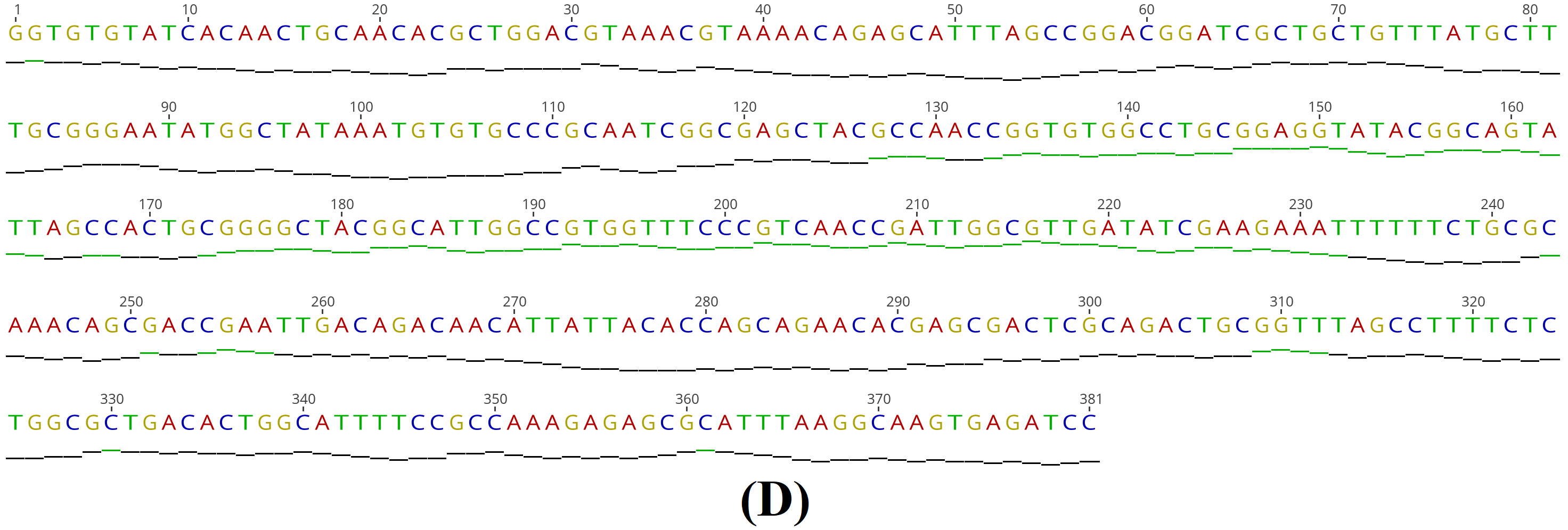


**Fig. S1.** Continued**.**


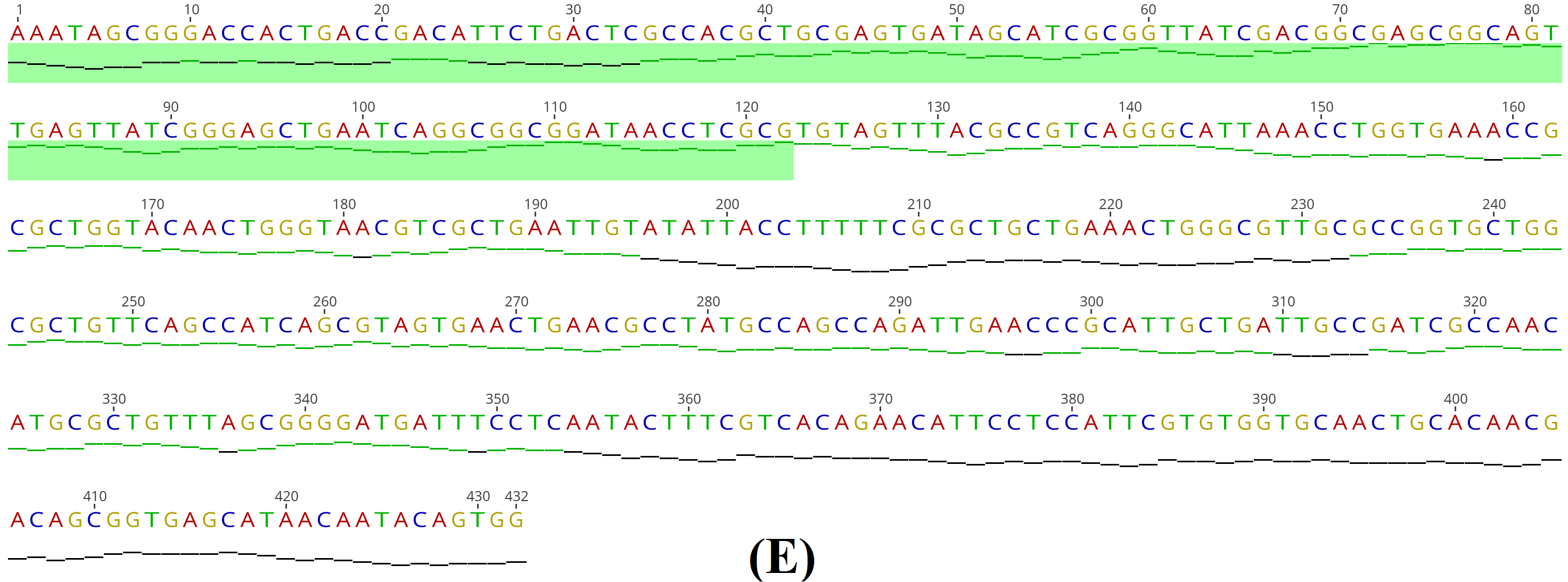


**Fig. S1.** Continued.


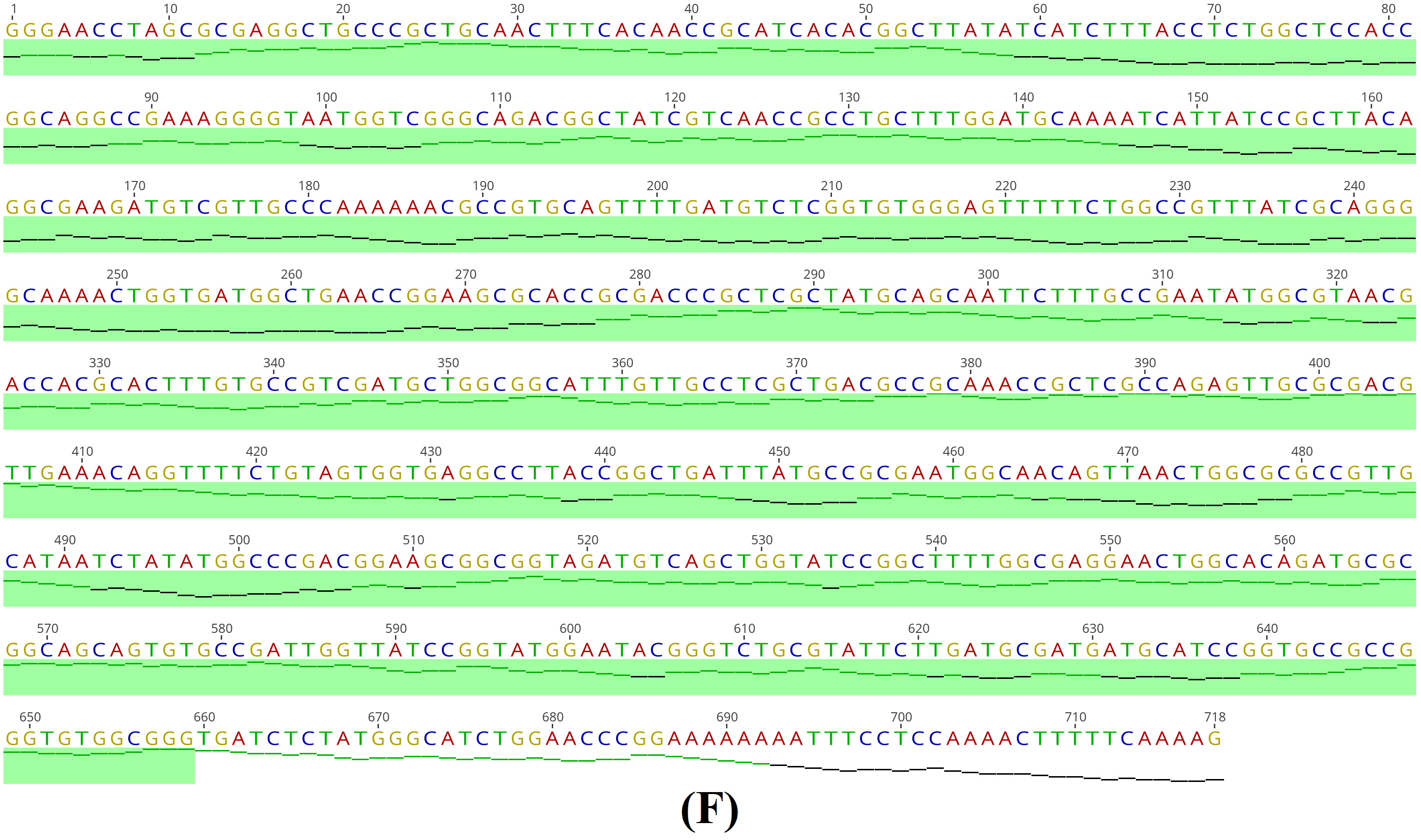
**Fig. S1.** Continued

**Table S1.** Identity matrix of *E. coli* OQ866153 enterochelin synthesis genes

|  | **OR645473, EntD** | **OW848980** | **LR134228** | **LT883142** | **LR134295** | **CP122704** | **LS483296** | **CP125073** | **LR881940** | **OR645470, EntA** | **OR645475-EntF** | **OR645474-EntE** | **OR645471, EntB** | **CP098739** | **OR645472, EntC** |
| --- | --- | --- | --- | --- | --- | --- | --- | --- | --- | --- | --- | --- | --- | --- | --- |
| **OR645473, EntD** | | 93.701 | 93.701 | 93.701 | 97.9 | 93.438 | 97.9 | 97.9 | 97.9 | 0 | 0 | 0 | 0 | 0 | 0 |
| **OW848980** | 93.701 |  | 97.058 | 95.977 | 94.585 | 97.443 | 97.094 | 75.45 | 98.165 | 33.806 | 32.179 | 35.976 | 21.25 | 23.178 | 33.085 |
| **LR134228** | 93.701 | 97.058 |  | 98.719 | 99.133 | 99.143 | 97.643 | 97.481 | 97.481 | 0 | 32.308 | 0 | 0 | 22.395 | 0 |
| **LT883142** | 93.701 | 95.977 | 98.719 |  | 98.981 | 98.834 | 97.797 | 97.462 | 97.42 | 0 | 32.308 | 0 | 0 | 22.121 | 0 |
| **LR134295** | 97.9 | 94.585 | 99.133 | 98.981 |  | 99.698 | 98.375 | 98.23 | 98.185 | 0 | 32.308 | 0 | 0 | 22.054 | 0 |
| **CP122704** | 93.438 | 97.443 | 99.143 | 98.834 | 99.698 |  | 98.063 | 98.24 | 98.215 | 0 | 32.308 | 0 | 0 | 22.571 | 0 |
| **LS483296** | 97.9 | 97.094 | 97.643 | 97.797 | 98.375 | 98.063 |  | 99.435 | 99.418 | 0 | 32.308 | 0 | 0 | 22.744 | 0 |
| **CP125073** | 97.9 | 75.45 | 97.481 | 97.462 | 98.23 | 98.24 | 99.435 |  | 99.951 | 0 | 32.308 | 6.467 | 0 | 19.566 | 0 |
| **LR881940** | 97.9 | 98.165 | 97.481 | 97.42 | 98.185 | 98.215 | 99.418 | 99.951 |  | 0 | 32.308 | 0 | 0 | 22.907 | 0 |
| **OR645470, EntA** | 0 | 33.806 | 0 | 0 | 0 | 0 | 0 | 0 | 0 |  | 0 | 0 | 0 | 91.136 | 0 |
| **OR645475-EntF** | 0 | 32.179 | 32.308 | 32.308 | 32.308 | 32.308 | 32.308 | 32.308 | 32.308 | 0 |  | 0 | 0 | 91.828 | 0 |
| **OR645474-EntE** | 0 | 35.976 | 0 | 0 | 0 | 0 | 0 | 6.467 | 0 | 0 | 0 |  | 0 | 95.718 | 0 |
| **OR645471, EntB** | 0 | 21.25 | 0 | 0 | 0 | 0 | 0 | 0 | 0 | 0 | 0 | 0 |  | 97.079 | 0 |
| **CP098739** | 0 | 23.178 | 22.395 | 22.121 | 22.054 | 22.571 | 22.744 | 19.566 | 22.907 | 91.136 | 91.828 | 95.718 | 97.079 |  | 98.258 |
| **OR645472, EntC** | 0 | 33.085 | 0 | 0 | 0 | 0 | 0 | 0 | 0 | 0 | 0 | 0 | 0 | 98.258 |  |
